# The ICES Indigenous Health Data Training Program: Building knowledge and strengthening capacity within Indigenous communities and organizations

**DOI:** 10.23889/ijpds.v9i5.2713

**Published:** 2024-09-18

**Authors:** Graham Mecredy, Jessica Jordao

**Affiliations:** 1 ICES

## Objective and Approach

The responsible use of population-level health data requires equitable access, and considerations of Indigenous data sovereignty. There is often a baseline level of knowledge and understanding required to access data. To facilitate data access and strengthen capacity within Indigenous communities and organizations, we created the ICES Indigenous Health Data Training Program (IHDTP).

## Results

The IHDTP provides learning opportunities for Indigenous communities and organizational members to gain a better understanding of ICES data with a focus on Indigenous health. It provides an opportunity to gain practical knowledge on the research process, including how to develop a research question, understanding and interpreting research outputs, and knowledge translation. The program offers ongoing one-on-one technical support to participants, as well as the opportunity to submit a research project request for participating communities and organizations.

## Conclusions

The IHDPT has been run several times, including both virtually and in-person, with a range of Indigenous partners. The materials presented in each administration of the program were tailored to the partner. Recorded sessions have also been created to allow a greater number of additional Indigenous partners to take the course flexibly. Program participants have indicated that the information learned would be used to advance issues that are important to their communities via policy, advocacy, and planning.

## Implications

The IHDTP has enabled a variety of Indigenous partners to strengthen their understanding of health data and research to ultimately facilitate equitable and inclusive public health data access.

